# The Forgotten Role of Central Volume in Low Frequency Oscillations of Heart Rate Variability

**DOI:** 10.1371/journal.pone.0120167

**Published:** 2015-03-20

**Authors:** Manuela Ferrario, Ulrich Moissl, Francesco Garzotto, Dinna N. Cruz, Ciro Tetta, Maria G. Signorini, Claudio Ronco, Aileen Grassmann, Sergio Cerutti, Stefano Guzzetti

**Affiliations:** 1 Department of Electronics, Information and Bioengineering (DEIB), Politecnico di Milano, Italy; 2 Fresenius Medical Care R&D, Bad Homburg, Germany; 3 San Bortolo Hospital, Vicenza, Italy; 4 L. Sacco Hospital, Università degli Studi, Milan, Italy; Université de Montréal, CANADA

## Abstract

The hypothesis that central volume plays a key role in the source of low frequency (LF) oscillations of heart rate variability (HRV) was tested in a population of end stage renal disease patients undergoing conventional hemodialysis (HD) treatment, and thus subject to large fluid shifts and sympathetic activation. Fluid overload (FO) in 58 chronic HD patients was assessed by whole body bioimpedance measurements before the midweek HD session. Heart Rate Variability (HRV) was measured using 24-hour Holter electrocardiogram recordings starting before the same HD treatment. Time domain and frequency domain analyses were performed on HRV signals. Patients were retrospectively classified in three groups according to tertiles of FO normalized to the extracellular water (FO/ECW%). These groups were also compared after stratification by diabetes mellitus. Patients with the low to medium hydration status before the treatment (i.e. 1st and 2nd FO/ECW% tertiles) showed a significant increase in LF power during last 30 min of HD compared to dialysis begin, while no significant change in LF power was seen in the third group (i.e. those with high pre-treatment hydration values). In conclusion, several mechanisms can generate LF oscillations in the cardiovascular system, including baroreflex feedback loops and central oscillators. However, the current results emphasize the role played by the central volume in determining the power of LF oscillations.

## Introduction

Since 1876, spontaneous human pulse and arterial pressure oscillations of frequency lower than that of respiration, also called “10-second rhythm”, have been the subject of numerous studies [10]. A variety of theories have been presented to explain the underlying mechanism, with the three main hypotheses focusing on central oscillations of sympathetic nervous system, baroreceptor reflex and peripheral oscillations theories [15][8].

Over the past twenty years, several experimental studies demonstrated an increase in the power of these low frequency (LF) oscillations during maneuvers or conditions characterized by an increase of sympathetic modulation [5, 7]. However, to date no definite conclusions could be drawn regarding the origin of these oscillations. A variety of unexpected observations stimulated a reconsideration of older theories. For example, the significant increase in LF oscillation power during passive standing in healthy subjects [12] was not observed during other motions of sympathetic activation, such as the prolonged handgrip [9]. Moreover, spectral analysis of heart rate variability [HRV) during exercise revealed that the magnitude of LF oscillations is influenced by body position, i.e. standing versus supine [13]. In addition, increased sympathetic activation in heart failure patients was found to be accompanied by a decreased LF variability component in RR interval and muscle sympathetic nervous activity (MSNA) [14][18][22].

This decrease in the power of these oscillations in patients with heart failure appears to be associated with increases in central volume. In fact, the low frequency power spectral component was reported to increase during all activities involving a decrease of central extracellular water, for example during passive tilt. In this study we tested the hypothesis that central volume plays a key role in LF oscillations using data from end stage renal disease (ESRD) patients undergoing dialysis. Depending on their compliance to diet and fluid intake prescriptions, dialysis patients can have very different levels of fluid overload (FO) when presenting for their next dialysis treatment. In addition, the dialysis treatment itself involves a large change in central blood volume, i.e. the amount of blood in the heart cavities, lungs and central arterial tree, and it is well-known that acute central hypovolemia induces sympathetic activation such that arterial pressure is maintained through systemic vasoconstriction and cardiac acceleration [3]. These variations in fluid shifts during dialysis treatment are larger than those considered in physiological models commonly referred to in the literature, e.g. lower body negative pressure (LBNP) protocol [4] or during blood donation.

The objective of the study is to provide further insight into the influence of central volume on LF oscillations in the power spectra of HRV, using data derived from a therapy (i.e. dialysis) that reduces central blood volume and thus triggers sympathetic nerve activation.

## Materials and Methods

### 1. Patient selection and Study design

Eighty chronic HD patients undergoing dialysis treatment at the San Bortolo Hospital, Vicenza, Italy were enrolled in this observational study. Inclusion criteria were age above 18 years and HD vintage of at least 6 months. Exclusion criteria were a HD frequency other than thrice weekly and hospitalization or antibiotic treatments in the preceding 8 weeks. The Institutional Review Board of San Bortolo Hospital approved the study. Patients signed informed consent prior to enrollment and the study was conducted in full accordance with the Declaration of Helsinki.

### 2. Measurements

A whole body bioimpedance spectroscopy method was employed to define the individual hydration state on the basis of an individual’s normal extracellular volume, taking into account the individual’s body composition. Fluid overload (FO) was assessed by the Body Composition Monitor (BCM, Fresenius Medical Care, Germany). This measures the impedance between 5 and 1000 kHz. High-frequency current passes through the total body water (TBW), whereas low-frequency current cannot penetrate cell membranes and thus flows exclusively through the extracellular water (ECW). ECW consists of the interstitial water, the plasma water and the transcellular water. Fluid status can thus be defined in terms of excess extracellular water using a physiologic model based on tissue hydration properties and taking body composition into account. The BCM provides the FO expressed in liters.

Whole body bioimpedance spectroscopy was conducted just before the midweek HD session for each enrolled patient. Twenty four-hour electrocardiogram (ECG) Holter recordings were started prior to the same midweek HD employing a three-lead Holter device with a sampling rate of 250 Hz (clickholter, Cardioline, et medical devices SpA, Italy).

The FO normalized over the extracellular water (FOpre/ECW%) was calculated from the absolute FO measured just before the HD session (FOpre). This index facilitates comparison between patients who may have the same FO value but a completely different distribution of fluids. In particular, FOpre/ECW% reflects the extent of central volume accumulation at the beginning of HD.

Patients were retrospectively classified in three groups according to the FOpre/ECW% tertiles. All analyses were repeated after exclusion of patients suffering from diabetes, a pathology that affects the autonomic nervous system.

As hematocrit (Hct) and plasma electrolytes changes can be related to volume variations, these were also considered in the study. Htc values were collected by means of a real-time online ultrasonic blood volume monitor (BVM, Fresenius Medical Care, Germany). In this device, variation of sound transmission velocity is proportional to changes in total protein content, i.e. sum of plasma proteins and hemoglobin. A model calculates Hct from these variations. Values of Hct were recorded every minute for each HD session, and were averaged during the first 10 minutes and the last 10 minutes of treatment (before priming volume reinfusion). Hct values reflect the circulating volume.

Finally, pre-dialysis plasma sodium, potassium and calcium concentrations were routinely measured on a monthly basis. As sodium is associated with fluid retention and thirst stimulus, these values (although not collected on the same day as ECG Holter analysis) were considered in the study.

### 3. HRV analysis

The R peaks were automatically identified and classified from the Holter ECG recordings (CubeHolter, Cardioline, et medical devices SpA, Italy). The obtained RR time series was subdivided into 5-minute epochs. Epochs with at least 85% of qualified sinus beats were considered and the so-called normal-to-normal (NN) intervals, i.e. all intervals between adjacent R peaks resulting from sinus node depolarization, were analyzed. Mean values of time domain and frequency domain were calculated for the first 15 minutes and the last 30 minutes of HD, to represent a baseline status (BL) and the final status after HD treatment, respectively.

The time domain parameters were: mean heart rate, the standard deviation of the NN intervals (SDNN), the square root of the sum of the squares of differences between adjacent NN intervals (RMSSD), the standard deviation of the averages of NN intervals in all 5-minute segments of the recording (SDANN), and the percentage of pairs of adjacent NN intervals differing by more than 50 ms in the sequence (pNN50).

The frequency domain parameters were calculated by means of the autoregressive power spectral analysis. The power in the very low frequency (VLF, 0.003<f≤0.04Hz), the low frequency (LF, 0.04<f≤0.15Hz) and the high frequency (HF, 0.15<f≤0.4Hz) bands were calculated, as well as the LF/HF ratio. The normalized power LF% was calculated as LF/(total power – VLF) × 100 and the normalized power HF% was calculated as HF/(total power – VLF) × 100.

### 4. Statistical analyses

One-way ANOVA or Kruskal–Wallis one-way analysis of variance was performed to identify differences between groups. Post-hoc comparisons were performed by Tukey's least significant difference (LSD) test.

Comparisons between the different HD epochs were performed in a paired fashion, by means of Wilcoxon signed rank test. Continuous variables are expressed as means ± standard deviation, or as medians with 25th and 75th percentiles. A p-value < 0.05, 2-tailed, was considered statistically significant.

## Results

Holter recordings and reliable BCM measurements were available for 69 patients who participated in the study. Further details on protocol are explained in Ferrario et al. [6]. Spectral analyses could not be performed for 11 of these patients because of a high incidence of arrhythmias in the baseline (first 15 min) or the final epoch (last 30 min) stages, bringing the final study population to 58. The patients were retrospectively divided into three groups according to FOpre/ECW% tertiles and compared, i.e. according to hydration status before the treatment. The corresponding patient characteristics are provided in Table 1. There were no significant differences among the three groups, with the exception of dialysis vintage. This was significantly higher in the third tertile group compared to both the first and the second groups.

**Table 1:** Data of analyzed patients.

	1st tertile	2nd tertile	3rd tertile
#	19	20	19
FO/ECW%	5.3 (1.6, 7.3)	13.2 (11.5, 15.8)	24.0 (19.0, 28.9)
Age [years]	62 (47, 75)	67 (60, 73)	70 (57, 77)
Gender [M/F]	11/8	15/5	10/9
Dyalisis Vintage [years]	4 (2, 6)	4 (2, 9)	9 (6, 11)*
Treatment time [min]	236 8234, 244)	236 (233, 239)	237 (233, 243)
UFR[L/hr]	0.67 (0.47, 0.84)	0.71 (0.32, 0.90)	0.81 (0.54, 0.89)
SBP [mmHg] at HD beginning	127 (120, 145)§	148 (140, 159)	142 (131, 151)
DBP [mmHg] at HD beginning	69 (65, 75)	75 (62, 84)	66 (61, 72)
SBP [mmHg] after 3hr HD	132 (123, 146)	153 (133, 163)	134 (130, 163)
DBP [mmHg] after 3hr HD	78 (68, 89)	75 (70, 86)	70 (64, 77)
Diabetes	6	5	3
PVD	9	8	8
CHF	2	3	5
LVH	14	13	14
Hypertension	19	19	18
beta-blockers	8	5	8
ACE inhibitors	7	6	6
Calcium antagonist	7	12	7

Values are expressed as median (25°,75° percentile) and the occurence of comorbidities and drug prescription are reported

* one-way ANOVA p<0.05, post hoc comparison p-value <0.05 vs 1^st^ tertile and 2^nd^ tertile

§ kruskall wallis ANOVA p<0.05, post hoc comparison p-value <0.05 vs 2^nd^ tertile.

SBP = systolic blood pressure; DBP = diastolic blood pressure.

Table 1 reports also the values of systolic blood pressure (SBP) and diastolic blood pressure (DBP) measured by the nurses at the beginning of HD and in the last HD period, i.e. at least after 3 hours of treatment. The initial values of systolic blood pressure resulted significant lower in the first group of patients, i.e. the ones with the lowest hydration values, with respect the values obtained from the second group. In all the groups there were no significant differences between initial and final values of both SBP and DBP.

SDANN and VLF during the last 30 min of HD were significantly lower in the third group (3^rd^ tertile) compared to the other two groups. Values of LF/HF during the last 30 min of HD were also significantly lower in the third group (3^rd^ tertile), but compared only to the first group (Kruskal–Wallis one-way analysis of variance p<0.05, post hoc comparison p-value <0.05). At baseline, VLF values in the second and third groups were significantly lower than in the first group at BL.

In comparisons of values at BL and during last 30 min of HD only the first two groups showed significant differences. The patients with the lowest hydration values (1st tertile) showed a significant increase in VLF, LF and LF/HF indices and a significant decrease in HF%. Similarly, the second group (2nd tertile) showed a significant increase in VLF and LF, but a slight increase in HF. No significant changes between BL and end of treatment were observed in the third group, i.e. the patients with high pre-treatment hydration values (Table 2).

**Table 2:** Values of time and frequency domain indices estimated during the first 15 min (baseline) and last 30 min of the HD session.

	1st FO/ECW% tertile			2nd FO/ECW% tertile			3rd FO/ECW% tertile		
	BL	last 30'HD	Δ = BL-last30'HD	BL	last 30'HD	Δ = BL-last30'HD	BL	last 30'HD	Δ = BL-last30'HD
**meanHR (bpm)**	66.2 (60.7, 74.7)	72.3 (60.4, 79.7)	2.1(−2.0,8.6)	69.0 (58.6, 78.9)	68.0 (61.3, 77.8)	2.1(−3.4,6.9)	67.6 (61.4, 73.4)	67.1 (61.1, 79.2)	0.5(−2.2,5.1)
**SDNN (ms)**	40.6 (30.6, 53.0)	43.5 (37.2, 71.7)	9.4(−3.3,21.4)	31.1 (25.9, 54.4)	58.6 (31.8, 80.6)	18.6(0.8,34.8)	25.9 (20.8, 46.4)	39.8 (24.1, 62.2)	5.0(−5.3,21.8)
**SDANN (ms)**	21.5 (6.1, 34.6)	**16.7 (13.9, 27.5)**	−2.3(−9.5,10.6)	12.6 (5.0, 21.2)	**14.5 (13.1, 50.8)**	4.2(−2.3,17.8)	8.6 (6.1, 11.7)	9.7 (6.7, 13.7)*°	1.6(−3.0,3.6)
**RMSSD (ms)**	895 (516, 1216)	871 (572, 1684)	90.0(−43.9,447.7)	1033 (505, 1567)	922 (561, 1513)	78.0(−388.0,380.7)	1143 (577, 1585)	759 (486, 1344)	50.4(−633.5,365.8)
**pNN50%**	1.02 (0.22, 3.64)	0.76 (0.33, 9.44)	0.04(−1.14,0.55)	0.90 (0.23, 6.36)	3.37 (0.63, 16.50)	0.41(−1.29,5.31)	1.02 (0.21, 4.45)	0.82 (0.09, 15.08)	0.02(−0.85,1.36)
**VLF (ms** ^**2**^)	**454 (317, 797)**	560 (362, 2157)	*227 (37,1308)#*	280 (127, 471)*	653 (269, 2042)	*278 (37, 1203)§*	233 (86, 376)*	320 (87, 721)	45 (−235, 317)
**LF (ms** ^**2**^)	72.7 (44.7, 348.8)	109.1 (43.8, 498.1)	*20.4 (2.3,184.1)#*	69.8 (30.1, 131.1)	205.5 (94.5, 653.5)	*85.4 (0.8, 237.8)#*	45.2 (18.1, 118.0)	42.6 (19.1, 303.9)	4.5 (−11.7, 101.9)
**HF (ms** ^**2**^)	46.6 (30.6, 169.1)	36.0 (9.0, 121.0)	−6.2 (−81.3,5.6)	39.3 (16.6, 55.1)	65.0 (31.6, 139.3)	*10.7 (−3.5, 60.0)#*	44.9 (21.4, 102.4)	45.8 (12.6, 126.3)	−2.8 (−36.0, 14.8)
**LF%**	32.9 (23.3, 41.0)	39.0 (26.6, 50.7)	6.8 (−0.7,14.7)	33.3 (16.3, 42.8)	41.6 (24.4, 51.1)	6.8 (−7.9, 16.1)	30.3 (18.8, 40.5)	25.6 (11.0, 47.2)	3.2 (−13.1,14.4)
**HF%**	19.0 (7.3, 27.5)	11.0 (5.4, 16.1)	*−4.9 (−14.3,−2.7)§*	14.9 (8.7, 29.6)	17.3 (6.4, 23.9)	3.4 (−6.0, 8.0)	35.2 (13.4, 45.3)	23.6 (6.4, 31.0)	−6.4 (−20.7,1.1)
**LF/HF**	1.67 (1.01, 3.03)	**2.64 (1.74, 7.17)**	*0.99 (0.80,2.72)§*	1.77 (0.76, 3.20)	2.81 (1.35, 6.05)	0.17 (−0.57, 3.28)	0.95 (0.57, 1.99)	1.68 (0.81, 2.42)*	0.26 (−0.23,1.31)

Patients are grouped in tertiles of pre-dialysis FO/ECW%.

Comparisons between baseline (BL) and last 30 min of hemodialysis (HD)

# Wilcoxon signed rank test p-value< 0.05, or

§ p-value<0.01 (italics)

*Kruskal–Wallis one-way analysis of variance p<0.05, post hoc comparison p-value <0.05 vs 1st tertile

°Kruskal–Wallis one-way analysis of variance p<0.05, post hoc comparison p-value <0.05 vs 2nd tertile

The bold values mark the indices, which significantly differ among groups.

**Fig 1:**
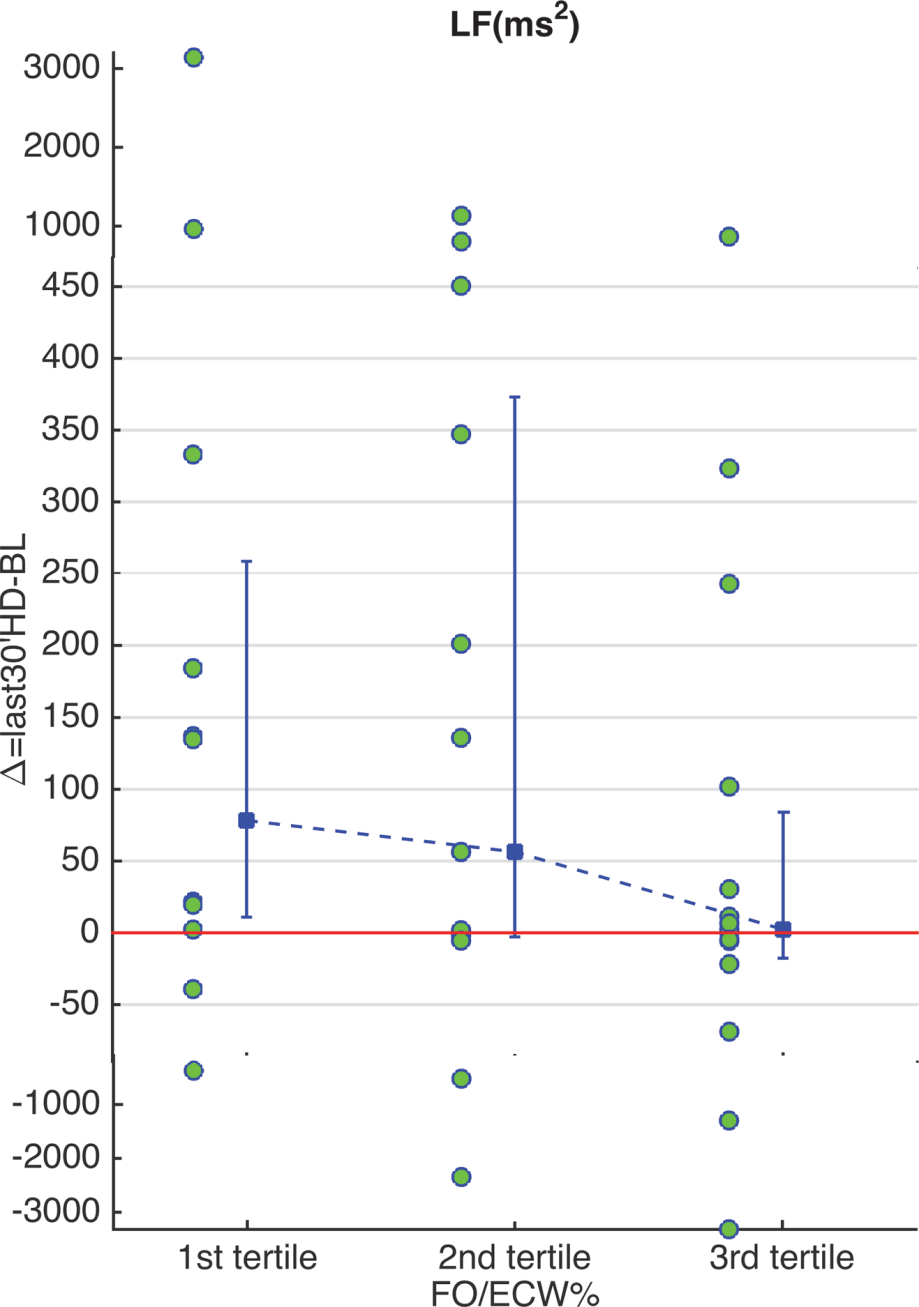
Values of the LF differences between baseline and last 30 minutes of hemodialysis. Values of the LF differences between baseline and last 30 minutes of hemodialysis in the three groups by excluding diabetes (see Table 3). The circles represent the singular patients. The errorbar represents the median and the 25^th^ and 75^th^ percentile (patients are grouped by the tertiles of FO_pre_/ECW% values). Note the increase in LF in the first tertile. The outliers are inserted in the picture at a different scale in order to focus the attention to the errorbars.

Results were similar after exclusion of diabetic patients (Table 3). Compared to BL, LF, LF% and LF/HF indices increased and HF% values decreased for patients with the lowest baseline hydration values (1st tertile). VLF and SDNN values increased in the second group (2nd tertile); no significant changes were observed in the third group. In particular, the patients with the highest hydration values (3^rd^ tertile) didn’t show any significant change in the LF oscillations (Table 3, Fig. 1).

**Table 3:** Values of time and frequency domain indices estimated during the first 15 min (baseline) and the last 30 min of the HD session.

	1st FO/ECW% tertile			2nd FO/ECW% tertile			3rd FO/ECW% tertile		
	BL	last 30'HD	Δ = BL-last30'HD	BL	last 30'HD	Δ = BL-last30'HD	BL	last 30'HD	Δ = BL-last30'HD
**meanHR (bpm)**	68.5 (60.1, 74.2)	72.3 (59.1, 80.2)	−1.1(−2.2,8.1)	66.6 (58.0, 79.2)	69.0 (61.3, 79.1)	2.2(−3.6,6.8)	68.2 (60.9, 74.1)	65.9 (61.2, 81.0)	−0.4(−1.9,4.9)
**SDNN(ms)**	41.2 (37.0, 54.8)	43.5 (37.7, 76.5)	2.9(−3.3,26.3)	35.0 (27.5, 51.6)	67.0 (38.5, 83.7)	*28.2(10.3,41.7)#*	27.1 (20.5, 50.7)	41.3 (26.5, 71.2)	3.1(−4.8,25.5)
**SDANN(ms)**	21.5 (13.9, 29.8)	19.7 (11.1, 36.7)	−4.1(−9.3,16.3)	12.3 (4.9, 17.6)	14.5 (12.5, 52.2)	6.3(−0.6,23.2)	9.5 (6.4, 12.9)	9.8 (7.1, 14.3)	−0.5(−3.0,3.2)
**RMSSD(ms)**	790 (477, 1228)	772 (523, 1628)	89.0(−45.3,347.5)	990 (483, 1598)	906 (561, 1501)	119.4(−388.0,380.7)	1171 (546, 1527)	724 (491, 1893)	53.1(−211.0,314.3)
**pNN50%**	1.13 (0.28, 3.13)	0.79 (0.22, 4.65)	0.00(−0.95,3.16)	0.77 (0.20, 6.36)	3.81 (0.61, 18.43)	0.55(−0.61,4.98)	0.72 (0.12, 5.18)	0.60 (0.11, 14.74)	0.00(−0.78,0.39)
**VLF (ms** ^**2**^)	640 (318, 833)	710 (355, 2177)	301 (92, 1374)	302 (64, 695)	795 (466, 2231)	*490 (10, 1548)#*	262 (95, 485)	342 (135, 657)	72 (−531, 280)
**LF (ms** ^**2**^)	122.9 (44.0, 391.8)	151.5 (53.1, 657.9)	*78.3 (10.9, 258.6)#*	52.3 (29.7, 189.3)	236.1 (73.6, 780.0)	56.4(−2.9,373)	45.8 (18.7, 142.7)	39.0 (19.3, 326.8)	2.4(−17.7,84.0)
**HF (ms** ^**2**^)	46.6 (23.0, 187.7)	43.8 (14.0, 118.2)	−4.1 (−89.2, 3.8)	37.3 (15.6, 71.8)	65.0 (32.7, 139.3)	9.9 (−29.8,41.7)	39.9 (18.9, 152.4)	30.7 (9.3, 121.6)	−4.5(−34.0,8.9)
**LF%**	26.5 (19.7, 44.7)	40.6 (32.6, 54.5)	*9.1 (2.2, 16.6)§*	26.2 (14.8, 41.5)	42.8 (21.2, 54.6)	3.7(−7.9,16.1)	30.9 (18.4, 41.6)	28.4 (11.6, 47.0)	5.1(−12.3,13.5)
**HF%**	14.1 (6.3, 25.0)	9.7 (3.8, 14.5)	*−4.3 (−10.7, −3.1)#*	14.5 (8.1, 30.2)	11.8 (5.3, 26.9)	−2.1(−6.0,8.1)	33.0 (10.8, 42.6)	20.4 (4.8, 34.6)	−4.4(−16.1,1.3)
**LF/HF**	1.79 (1.17, 3.25)	4.04 (2.32, 8.63)	*1.7 (0.8, 6.4)§*	1.86 (0.37, 3.51)	3.55 (1.23, 6.14)	0.2(−0.6,3.7)	1.04 (0.58, 2.36)	1.88 (0.98, 3.05)	0.38(−1.12,1.80)

Patients are grouped in tertiles of pre-dialysis FO/ECW%. Diabetic patients are excluded.

Comparisons between baseline (BL) and last 30 min of hemodialysis (HD)

# Wilcoxon signed rank test p-value< 0.05, or

§ p-value<0.01 (italics)

**Fig 2:**
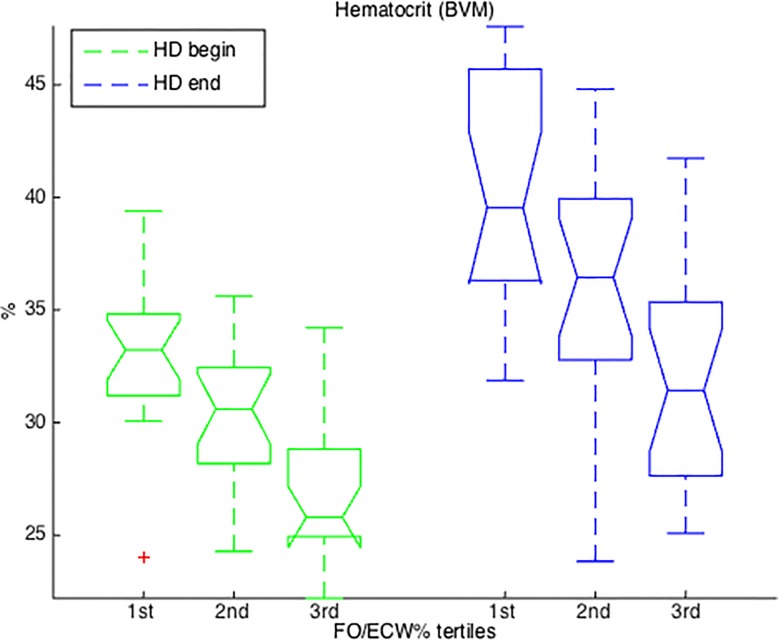
Hematocrit values. Boxplot of patient hematocrit values at the start and end of hemodialysis grouped by pre-dialysis FO/ECW% tertiles. HD: hemodialysis. BVM: Blood Volume Monitor device.

A comparison of Hct values revealed significant differences between groups (Kruskal–Wallis one-way analysis of variance p-value<0.0001), both at the beginning and at the end of the treatment. Values in the third group were lower than in the other two groups (post-hoc comparisons p-value<0.05) (Fig. 2).

Serum sodium values did not differ between groups. However, values in the third group tended to higher (1st tertile: 137 (135, 139) mEq/L; 2nd tertile: 137 (136, 139) mEq/L; 3rd tertile: 139 (137, 141) mEq/L; Kruskal–Wallis one-way analysis of variance p-value = 0.13).

## Discussion

An association between LF power and pharmacological sympathetic modulation was reported in 1981 by Askerlod et al [1] in an animal experiment, and was confirmed by a number of experimental and clinical studies over the following decades. In the present study, we observed that values of SDANN and LF/HF during the last 30 min of HD were significantly lower in the third FOpre/ECW% tertile group (i.e. patients with high pre-dialysis hydration values) compared to the first FOpre/ECW% tertile group (Table 2). Moreover, the patients with low or medium pre-dialysis hydration status (i.e. 1st and 2nd tertiles) showed a significant increase in LF power during last 30 min of HD compared to baseline, while this did not change significantly in the third group (i.e. patients with high pre-dialysis hydration values) (Table 2).

The diabetic condition per se does not affect the HRV. However, diabetic patients often suffer from cardiovascular autonomic neuropathy (CAN), a pathology characterized by a generally reduced HRV [23]. Some works demonstrated that some diabetic patients who had not yet been clinically diagnosed with CAN [2] showed a significantly reduced sympathetic response to autonomic tests. By excluding the diabetic patients from the analyses, who may be at risk of CAN, we see that the initial observations still hold (Table 3, Fig. 1).

These results allow a more differentiated interpretation of low frequency oscillations. In patients with lower hydration level, a reduction in central volume due to dialysis leads to a reactivation of the capacity of the cardiovascular system to oscillate at low frequency (LF). However, for dialysis patients with higher hydration status, the same removal of fluid during dialysis does not reduce the central volume sufficiently to reactivate this oscillatory frequency. This does not exclude contributions of the sympathetic nervous system, baroreceptors and general autonomic modulation in the regulation of LF power. However, we believe that these could play just a reinforcement (entrained) role in the direct action of volume on vascular structures [20]. If heart rate LF oscillations would be primarily the result of autonomic response (e.g. baroreceptor mediated) to hemodynamic perturbations (as induced by dialysis treatment), we would expect to see a significant and clear increase of LF power at the end of dialysis also in the third tertile group.

Three different types of studies indirectly support this hypothesis and underline the capacity of the peripheral vascular system to oscillate independently from heart beat and neural control.

1. Studies of animals with simple vascular systems, such as insects. These animals have a compact heart for intensive pumping of insect “blood” (hemolymph) into the head, the thorax and flight musculature. However, the visceral organs of these insects exhibit myogenic, extracardiac peristaltic pulsations similar to a slower heart beat [21][24].
2. Studies on the embryonic human heart. Here, unidirectional blood flow is observed even in the absence of valves. This is achieved by the pumping action of the embryonic heart tube, which is driven by traveling oscillatory mechanical waves sweeping from the vascular venous to vascular arterial end [11].
3. Experimental studies with small cell groups. Such studies demonstrated how an intrinsic oscillation in membrane potential of a single smooth muscle cell can become synchronized with the oscillations of other cells, such that large populations of cells may oscillate synchronously and cause oscillations in resistance to flow and/or capacitance [19].

The results of our study provide valuable insight into the interpretation of other study results. For example, the large increase in the power of LF oscillations observed during active or passive standing in healthy subjects [12] was not reported during other sympathetic activation activities in which central volume was unchanged, like the prolonged handgrip [9]. Also, the magnitude of LF oscillations observed during exercise was reported to be influenced by body position, i.e. standing or supine positions—both of which are characterized by sympathetic activation [13]. Another example: the LF variability component in RR interval and MSNA is decreased in heart failure patients despite increased sympathetic activation [22]. One can interpret this as being due to the increased central volume present in this pathological condition.

In parallel to our findings, Ramchandra et al. [17] reported that both volume expansion and hemorrhage caused no change in cardiac sympathetic nervous system (SNA) in heart failure sheep. Also, a reduction of FO was previously shown to be associated with a decrease of LF oscillations in a small group of patients starting with persistent and clear fluid overload [6].

### Limitations

This study had some limitations. The sample size was insufficient to allow consideration of all possible confounding factors, e.g. medication types. Also, further studies are needed to investigate long term effects of central volume changes on HRV LF oscillations, for example after fluid status normalization in high FO heart failure patients treated with diuretics or by dialysis.

## Conclusion

In conclusion, LF oscillations in the cardiovascular system can be generated by a variety of mechanisms, including baroreflex feedback loops [8] and central oscillators [16]. The results of this study underline the key role played by central volume in determining the power of LF oscillations. These results reinforce the opinion that interpreting LF oscillations as simply the result of sympathetic outflow is misleading. Assessment of patient hydration status facilitates a more comprehensive analysis of HR oscillations and a better understanding of patient response to therapies/activities that involve changes in the patient’s central volume, such as diuretic use or ultrafiltration in congestive heart failure patients.
